# Metal-Modulated Growth of Cubic, Red-Emitting InGaN Layers and Self-Assembled InGaN/GaN Quantum Wells by Molecular Beam Epitaxy

**DOI:** 10.1021/acsaelm.4c02174

**Published:** 2025-02-28

**Authors:** Silas A. Jentsch, Mario F. Zscherp, Vitalii Lider, Fabian Winkler, Andreas Beyer, Jürgen Belz, Nicolai M. Gimbel, Markus Stein, Donat J. As, Anja Henss, Kerstin Volz, Sangam Chatterjee, Jörg Schörmann

**Affiliations:** † Institute of Experimental Physics I and Center for Materials Research, Justus Liebig University Giessen, Heinrich-Buff-Ring 16, D-35392 Giessen, Germany; ‡ Materials Sciences Center and Faculty of Physics, Philipps-University Marburg, Hans-Meerwein-Strasse 6, D-35032 Marburg, Germany; § Department of Physics, Paderborn University, Warburger Strasse 100, D-33098 Paderborn, Germany

**Keywords:** molecular beam epitaxy, In_*x*_Ga_1−*x*_N, metal-modulated epitaxy, cubic III-nitrides, quantum wells, TEM, optical properties, SIMS

## Abstract

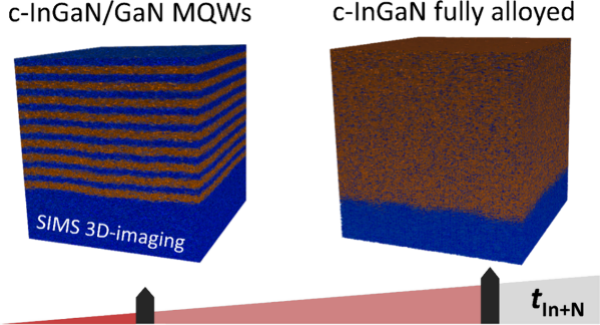

Cubic InGaN alloys are a promising candidate material for next-generation optoelectronic applications as they lack internal fields and promise to cover large parts of the electromagnetic spectrum from the deep UV toward the mid-infrared. This demands high-quality epitaxial growth of cubic InGaN/GaN quantum wells, especially for the red energy range. However, the growth of indium-bearing nitride quantum wells in the metastable cubic phase still poses many challenges. InGaN and GaN are typically grown at different temperatures and with different metal fluxes in molecular beam epitaxy, leading to either long waiting periods for temperature adjustment or growth under suboptimal conditions. Both degrade the crystal quality and optical properties. In this study, we apply a metal-modulated growth approach in molecular beam epitaxy that enables us to grow either self-assembled, phase pure, cubic InGaN/GaN multi quantum wells (MQWs) or homogeneous c-InGaN layers, only by adjusting the shutter duration times for Ga and In. We achieve smooth surfaces and sharp interfaces with a quantum well thickness tunable from 6 to 16 nm and a barrier thickness ranging from 4 to 10 nm. X-ray diffraction confirms >99% phase purity of our cubic layers, while time-of-flight secondary ion mass spectrometry, scanning transmission electron microscopy, and energy-dispersive X-ray spectroscopy provide detailed information on the quantum well composition and strain. Photoluminescence measurements at room temperature demonstrate the emission properties of the samples, with the emission wavelength ranging from 540 to 670 nm. Changing the barrier and QW thickness results in a shift of emission energy of up to 400 meV, which is explained by quantum confinement and strain. The high interface quality and excellent optical properties of the quantum wells without the need for multiple metal sources or long waiting times represent a significant advance in the development of next-generation optoelectronic devices.

## Introduction

Efficient light sources such as light-emitting diodes (LEDs) are in great demand: currently, 15–20% of the global electricity consumption is attributable to lighting applications.^1,2^ The state-of-the-art active material for red LEDs is (Al,Ga,In)P, while blue, green, and white LEDs commonly use hexagonal nitrides. The (Al,Ga,In)P system exhibits a transition to an indirect bandgap by changing the composition toward higher emission energies,^3−5^ making it unsuitable for green to blue emission. In contrast, the emission energies of hexagonal nitrides with (Ga,In)N/GaN multi quantum well (MQW) structures as active structures can be tuned from the ultraviolet across the visible spectrum to the infrared with increasing indium content. Unfortunately, this alloying increases the polar character of the wurtzite-structured hexagonal nitrides.^6,7^ The emerging internal electric fields reduce the overlap of the electron and hole wave functions.^8^ This quantum confined Stark effect (QCSE) inherently reduces the radiative recombination rates.^9^ It worsens with increasing indium content toward longer wavelength, i.e., green to red emission.^10,11^ In the case of micro-LEDs, these reduced radiative recombination rates become even larger, with efficiencies of around approximately 3% for a red 4 μm LED device.^12^ Micro-LEDs promise to revolutionize the landscape of digital displays and illumination, offering advantages in terms of brightness, lifetime, color, and minimum pixel size compared to liquid-crystal or organic LED displays.^13^ Here, nitrides show great potential, as they lose less efficiency when reducing the device size compared to phosphides.^14,15^

Cubic nitrides are promising candidate material systems with the potential to address efficiency challenges in micro-LEDs, particularly in the red spectral range, within one single material platform. Most importantly, this metastable phase does not exhibit internal fields due to its zincblende lattice structure.^16,17^ They also feature a smaller bandgap energy compared to the hexagonal structures. Thus, around 10% less indium incorporation is needed here for any given target wavelength in the visible range. These advantages have rekindled the research efforts toward cubic nitrides.^18−21^ However, significant challenges remain as, for example, the metastability limits their growth window and the lack of suitable, high-quality substrates leads to a high defect density.^20^ Furthermore, hexagonal inclusions^22,23^ occur as the indium content increases.

The extreme nonequilibrium conditions in molecular-beam epitaxy (MBE) are advantageous to grow cubic InGaN. However, the growth of cubic InGaN/GaN quantum wells in itself poses challenges beyond the issues related to the metastable phase.^18,24−26^ The growth of high structural quality gallium nitride and indium gallium nitride requires very different growth temperatures.^26−28^ Consequently, either the growth temperature is changed between well material and barrier growth or the growth is performed at the same growth temperature. The former can lead to unintentional defects during the associated waiting times,^24,29^ while the latter results in poorer crystal quality accompanied by inferior optical properties.^30^ Another related challenge is the need for different gallium fluxes for the optimal, metal-rich MBE growth of InGaN and GaN. For MBE systems with only one gallium source, this either mandates changing the temperature of the gallium cells between well and barrier growth or growth under suboptimal growth conditions with the same gallium flux. Achieving sharp interfaces and smooth surfaces is another important goal for the fabrication of InGaN quantum wells. Increased interface and surface roughness have a negative effect on the light-emitting performance of the MQWs.^31−35^

In this work, we explore the application of a metal-modulated growth scheme in molecular beam epitaxy for the growth of cubic InGaN. Our approach enables both the growth of homogeneous c-InGaN layers and self-assembled c-InGaN/GaN QWs. We achieve phase pure cubic layers with an indium content of 27% and a total thickness of 160 to 200 nm without changing the gallium cell or growth temperature during the quantum well growth. Furthermore, we show the tunability of well and barrier thickness, smooth surface, and sharp interfaces for the quantum wells and the optical response of all samples. All of these properties are paramount for prospective optoelectronic applications.

## Experimental Section

A metal-modulated growth approach in plasma-assisted molecular beam epitaxy is used to grow cubic In_*x*_Ga_1–*x*_N layers and cubic In_*x*_Ga_1–*x*_N/GaN quantum wells. The total thickness of these layers is approximately 160 to 200 nm with an indium content of 27% for samples A–C and a slightly lower content of around 17–21% for sample D according to X-ray diffraction. The 600 nm thick c-GaN templates are grown on 8 nm thin c-AlN buffers on commercial 3C-SiC/Si(001) pseudosubstrates from NovaSiC.^36^ The samples are grown in a Riber Compact12 MBE chamber with standard effusion cells under a constant beam equivalent pressure of 1.8 × 10^–7^ mbar and 1.9 × 10^–7^ mbar for gallium and indium. An Oxford Applied Research HD25 radio frequency plasma source supplies the nitrogen at a flow rate of 0.7 sccm and 200 W. All cubic InGaN layers and InGaN/GaN QWs are grown in metal-modulated growth mode at a constant substrate temperature of 600 °C, which is measured with a pyrometer. The gallium and indium shutters are alternately opened 10 times each with short intervals in between when both shutters are closed. The nitrogen source is constantly open. The four samples discussed in this work differ in *t*_In+N_, i.e., the time span of suppling both indium and nitrogen, which varies between 280 and 50 s. *t*_Ga+N_ and *t*_break_ have been set to 280 and 5 s, respectively.

Time-of-flight secondary ion mass spectrometry (ToF-SIMS) investigations were conducted using a M6plus instrument (IONTOF GmbH) equipped with a 30 kV Bi liquid metal ion gun (LMIG) for analysis, a dual source column (DSC), and a gas cluster source (GCIB) for depth profiling. Depth profiles were recorded in non-interlaced mode with Cs^+^ as sputter species at an energy of 500 eV and with Bi^+^ clusters for analysis. Each analysis frame followed a sputter step with a fluence of approximately 1.6 × 10^16^ ions/cm^2^ after a pause of 0.5 s. The LMIG current was 0.4–1.4 pA with a cycle time of 80 μs. The size of the sputter crater was 300 × 300 μm^2^, and the analysis field was 75 × 75 μm^2^ with 128 × 128 pixels. The measurements were performed in negative polarity in the spectrometry mode of the LMIG and in the all-purpose mode of the analyzer, achieving a mass resolution *m*/Δ*m* > 13,000 @ *m*/*z* = 82.93 (GaN^–^). An electron flood gun was used for charge neutralization. The spectra were calibrated to C^–^, C_2_^–^, and C_3_^–^, and the data were evaluated with SurfaceLab 7.3 (IONTOF GmbH).

Reciprocal space maps (RSMs) of the (002) and (−1–13) cubic nitride reflection were performed with high-resolution X-ray diffraction (HRXRD) on a Rigaku SmartLab diffractometer operating with a 9 kW rotating Cu anode.

The scanning transmission electron microscopy (STEM) measurements of focused ion beam prepared electron transparent cross-section lamellas were performed using a double aberration-corrected JEOL JEM-2200FS microscope with an operation voltage of 200 kV and an annular dark-field (ADF) detector. The high-angle ADF (HAADF) imaging mode was used providing so-called Z-contrast.^37^ In addition, energy-dispersive X-ray spectroscopy (EDX) measurements using an XFlash 5060 system were carried out for determination of the local indium concentration. The standardless quantification is based on calculated Cliff–Lorimer factors^38^ using Bruker Esprit 2.3 with background and peak deconvolution processing within the software.

For photoluminescence measurements, the samples have been excited at room temperature with a 405 nm laser diode using an excitation power of *P*_exc_ = 16.7 mW. For detection, a cooled, back-illuminated CCD camera has been used.

## Results and Discussion

**Figure 1:**
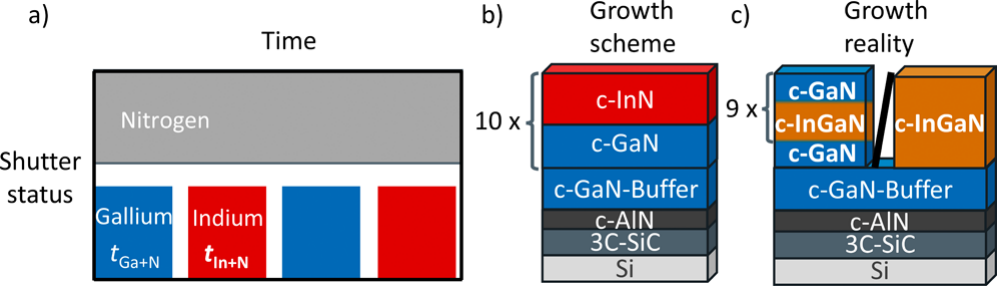
(a) Illustration of the shutter timing scheme used for the metal-modulated c-InGaN growth. The indicated time parameters *t*_Ga+N_ and *t*_In+N_ refer to the time with open nitrogen plus gallium or indium shutter, respectively. Illustration of the sample setup with (b) the nominal layer sequence of the metal-modulated growth with 10 growth cycles of *t*_Ga+N_ plus *t*_In+N_ and (c) the cases of the actual grown layers of either 9 c-InGaN/c-GaN quantum well layers or fully alloyed c-InGaN layers.

Modulated shutter operation in molecular beam epitaxy facilitates the high-quality growth of cubic InGaN and InGaN/GaN MQWs on c-GaN/c-AlN/3C-SiC/Si templates.^36^ The shutter timing and, thus, the material’s supply for this growth mode are illustrated in Figure 1a: the nitrogen shutter is continuously open, and we alternatingly open and close the indium and gallium shutter, ensuring a small intermediate “growth interruption” when both are closed. Naively, such a shutter sequence infers the deposition of a c-InN/c-GaN superlattice (SL) (Figure 1b). For the thermodynamically stable hexagonal phase with a similar shutter sequence, few monolayer thick InGaN/GaN superlattices were observed.^28,39^ For the cubic systems, though, we observe fully alloyed c-InGaN layers and c-InGaN/InGaN or c-InGaN/GaN superlattices depending on the indium shutter time *t*_In+N_; however, we never deposit c-InN (Fig 1c). Consequently, we explore the impact of *t*_In+N_ on nanostructure formation and its photoluminescence properties to elucidate the growth mechanism and thereby derive design opportunities for future LED applications.

**Figure 2:**
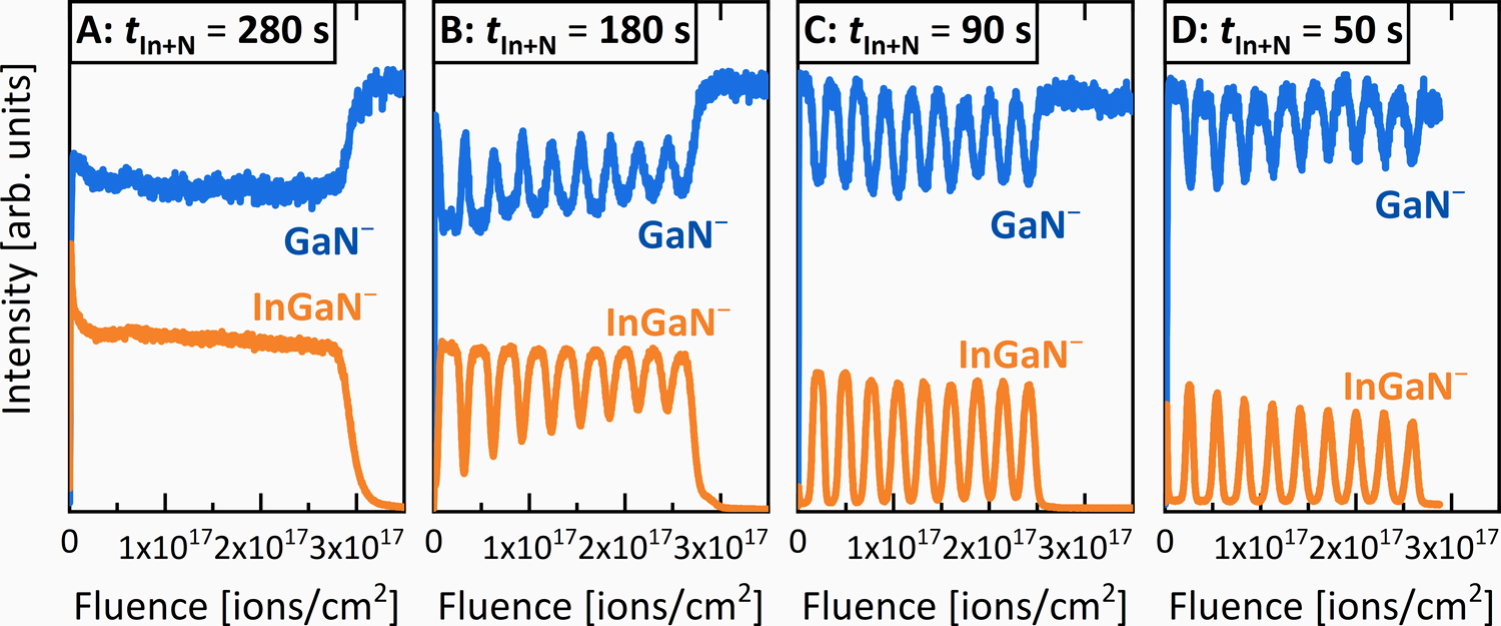
ToF-SIMS depth profiles showing the course of the GaN^–^ (blue) and InGaN^–^ (orange) signal for samples A–D with different *t*_In+N_.

ToF-SIMS resolves depth profiles over a large area, ensuring good comparability with X-ray diffraction. Figure 2 presents depth profiles of the GaN^–^ and InGaN^–^ cluster intensity from the surface to the InGaN–GaN interface for samples A–D with different *t*_In+N_ values between 280 and 50 s. Sample A (*t*_In+N_ = 280 s) shows a homogeneous InGaN^–^ profile without indium content fluctuations. This reveals the fully alloyed composition of the InGaN layer. For sample B (*t*_In+N_ = 180 s), clear interfaces are visible in the SIMS profile. The higher InGaN^–^ plateaus are referred to as wells, and the layers with a lower InGaN^–^ intensity as barriers. The GaN^–^ intensity of these barriers is lower than the intensity of the GaN buffer layer. Thus, the barriers consist of ternary InGaN with a lower indium content instead of binary GaN. Therefore, sample B is an InGaN/InGaN superlattice with alternating indium content layers. In addition, the SIMS data infer a concentration gradient in the layers with a lower indium content. Clear interfaces are visible for samples C (*t*_In+N_ = 90 s) and D (*t*_In+N_ = 50 s). For both, the GaN^–^ intensity of the barrier does align with the GaN buffer level, inferring InGaN/GaN superlattices.

The formation of fully alloyed InGaN layers for longer *t*_In+N_ and superlattices for shorter *t*_In+N_ can be explained with indium accumulation at the surface during *t*_In+N_. During this shutter interval, the epitaxial growth of InN is hindered because InN decomposes at substrate temperatures above 500 °C.^40,41^ Consequently, actual epitaxial growth takes place during the following *t*_Ga+N_. Here, the indium that is present at the surface and the additionally supplied gallium and nitrogen from the gas phase form a c-InGaN layer on the crystal surface. This growth mechanism infers the exchange of gallium atoms through the metallic indium layer on the surface to the semiconductor surface. This vertical cation segregation (VCS) is established for AlGaN formation.^42^ The larger cation species, in our case indium for InGaN, tends to diffuse away from the epitaxial growth surface and gets replaced by the smaller cation, in our case gallium. As the growth of InGaN progresses, the reservoir of indium on the surface decreases. Once the indium reservoir at the surface is depleted, binary GaN forms. In the case of sample A, sufficient indium remains at the surface after 280 s of *t*_In+N_, such that InGaN is formed during the entire *t*_Ga+N_, leading to the fully alloyed sample A. In contrast, only a smaller amount of indium is provided in sample D (*t*_In+N_ = 50 s) and is rapidly consumed in the following *t*_Ga+N_. Once the surface is indium-free, pure GaN forms, resulting in this self-assembled InGaN/GaN superlattice. When counting the interfaces of samples B–D in the SIMS data, it is noticeable that only 9 InGaN layers are visible despite the growth of 10 cycles. This is because one cycle begins with *t*_Ga+N_ and is then followed by *t*_In+N_. The consequence is that the last indium step is not followed by a gallium sequence, leading to one InGaN layer less than cycle numbers.

The indium content measured by (−1–13) reciprocal space maps is around 27% for samples A–C (Figure S1). This matches the indium content of conventionally grown epilayers at identical growth temperatures.^19^ For sample D featuring the shortest *t*_In+N_, the indium content cannot be determined reliably through RSMs due to the occurrence of superlattice satellite peaks instead of a single c-InGaN peak. Modeling the oscillation period of this sample suggests a lower indium content of *x*(In) = 0.17–0.21. These observations infer that the indium content is independent of *t*_In+N_ until the supplied amount of indium is insufficient, and interfacial composition gradients reduce the average indium content.

**Figure 3:**
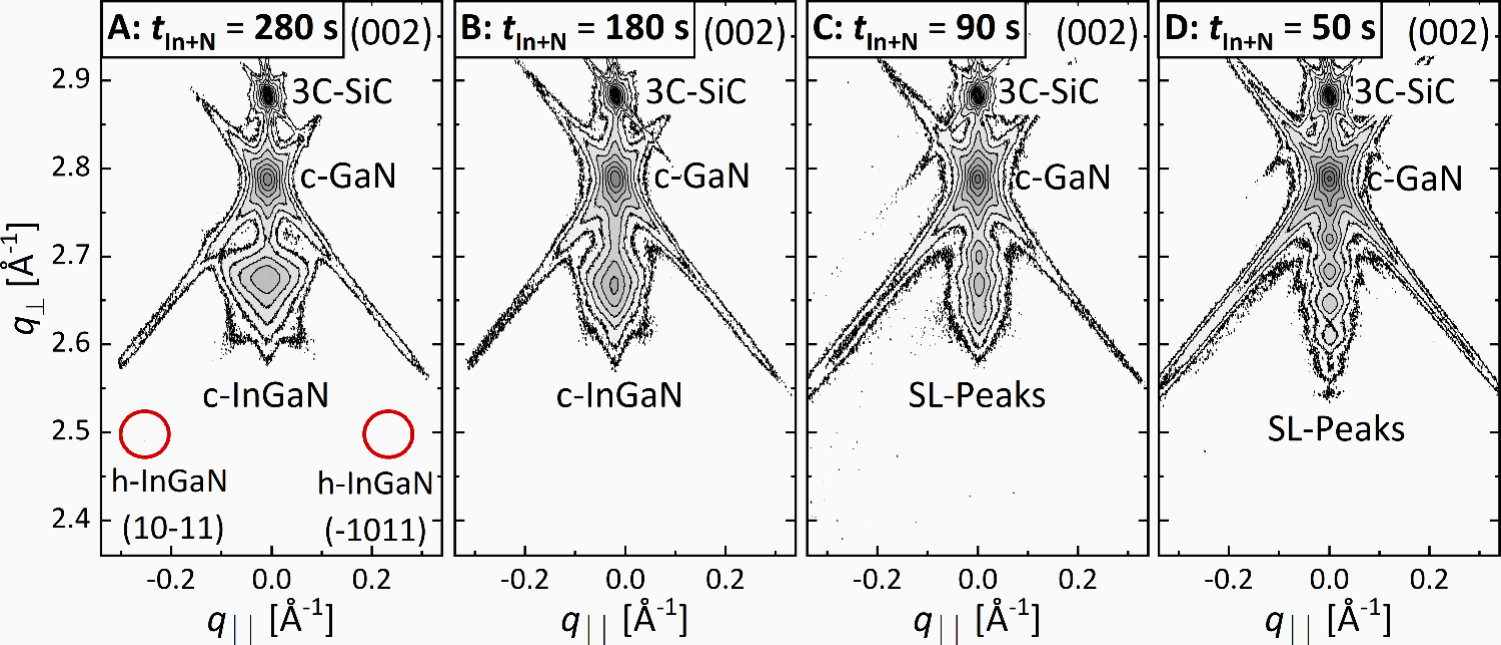
Reciprocal space maps around the (002) diffraction peak of c-GaN for samples A–D with different *t*_In+N_.

RSMs verify the high cubic phase purity of over 99% for all samples. This is evident in Figure 3, which presents the (002)-RSMs of samples A–D. No hexagonal (10–11) and (−1011) reflections are visible for any of the samples; these would appear in the regions indicated by the red circles in Figure 3. The (002)-RSMs demonstrate the transition from a fully alloyed c-InGaN layer to a superlattice, indicated by multiple satellite peaks, with a decreasing *t*_In+N_. Samples A and B feature only one single c-InGaN peak. This is expected in the case of sample A, as a fully alloyed layer is also observed by SIMS. In contrast, the ToF-SIMS depth profile of sample B indicates the presence of a superlattice (Figure 2). However, the profile also suggests a gradual change of the composition of the c-InGaN barriers, which would explain the absence of satellite peaks in Figure 3. Samples C and D show clear satellite peaks with peaks up to the sixth order for sample D. Satellite peaks only occur at superlattices with an excellent thickness and composition homogeneity as well as high structural quality of the interface.^43,44^ Asymmetric RSMs of the c-GaN (−1–13)-reflection reveal highly strained c-InGaN layers (Figure S1). We assign this strain an important role in achieving phase purity, as strain is known to stabilize the crystal phase and prevent phase separations.^19,45−47^ This high quality of the interfaces combined with the phase purity is a necessary requirement for efficient light emission of cubic InGaN quantum wells in future LEDs.^31−35^

**Figure 4:**
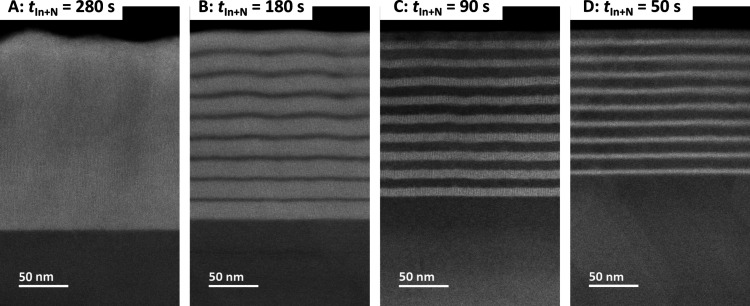
STEM HAADF images of samples A–D with different *t*_In+N_ revealing the homogeneous InGaN layer for sample A and MQW structures with different layer thicknesses for samples B–D.

Analyzing the samples by scanning transmission electron microscopy reveals the microscopic structural quality of the layers beyond the SIMS and X-ray diffraction (XRD) results. Figure 4 compares HAADF images of cross sections of samples A–D. The image of sample A consists of one homogeneous, fully alloyed layer with a rough surface. Imaging of samples B–D confirms smooth quantum well structures and shows a decrease in c-InGaN layer thickness with decreasing *t*_In+N_. These results match perfectly with the SIMS and XRD data. The transformation from a rough surface to a smooth morphology can also be observed in the atomic force microscopy images shown in Figure S1. The thickness of the layers varies for samples B to D between 16 and 6 nm for the well thickness and 4 and 10 nm for the barrier thickness. This shows that the well and barrier thickness can easily be tuned by simply changing *t*_In+N_. In combination with the smooth surface, this is an important characteristic of the growth of quantum wells. In general, the interfaces appear to be sharper near the c-GaN template and less so toward the surface. The blurred interfaces in the upper layers may originate from accumulative interface roughness with rising layer thickness.^25,48^ However, the blurring is more likely just an effect of projection through a higher TEM sample thickness due to a slight thickness gradient of the lamella.

**Figure 5:**
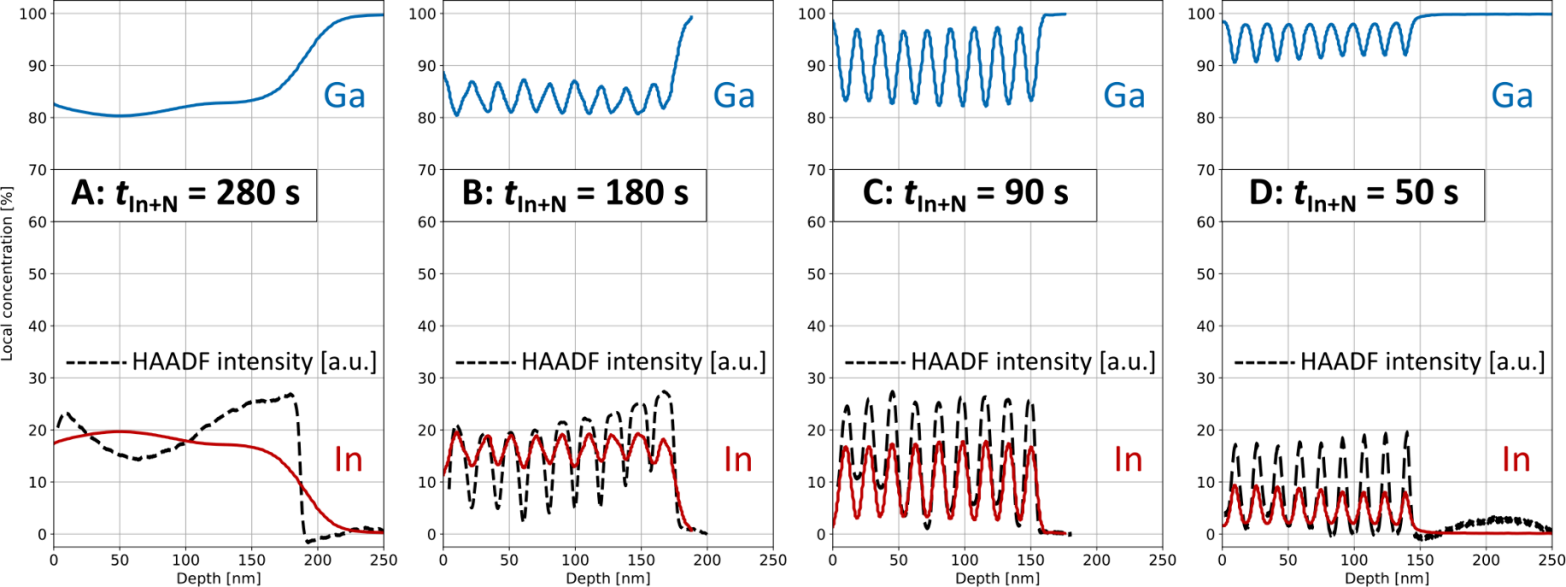
Averaged line profiles of quantitative EDX measurements showing the local concentration of In and Ga of samples A–D with different *t*_In+N_. The black dotted line shows the HAADF intensity derived from the STEM images.

Quantitative EDX was performed to assess the local indium content. Figure 5 presents averaged EDX line profiles for all four samples as well as the HAADF intensity. The EDX results generally support those of ToF-SIMS and RSM. The interface abruptness and concentration are reduced by the transmission through a finite sample volume and the imminent scattering and projection mixing effects. The maximum indium intensity of the EDX scans suggests that the composition of the c-InGaN QWs is indeed very similar for samples A and B, whereas *x*(In) appears to be slightly lower for sample C and significantly lower for sample D. This correlation is in good agreement with the XRD data and simulation. However, the maximum indium concentration, approximately 20%, is lower than the values obtained from XRD measurements. The difference is within the error range of the EDX and more pronounced for the thin layers due to a greater interface mixing of wells and barriers during transmission.

**Figure 6:**
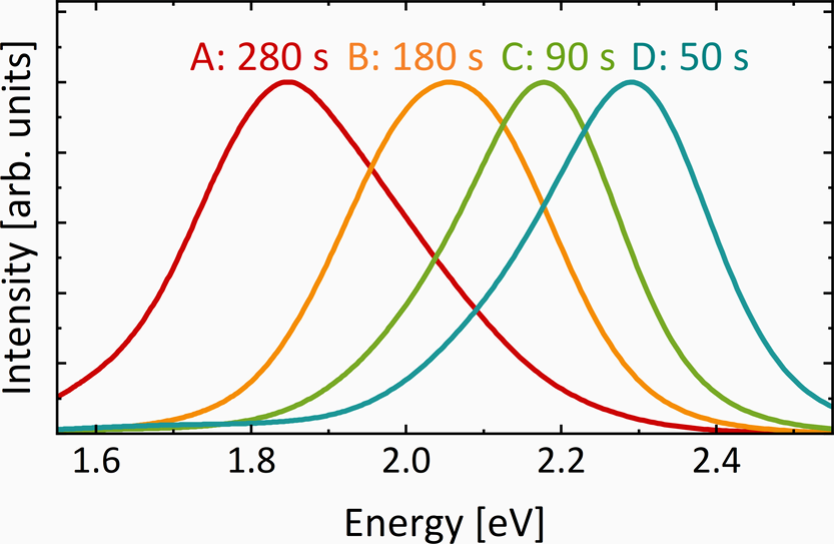
Normalized photoluminescence spectra at 290 K of samples A–D with different *t*_In+N_.

After the structural properties are clarified, we investigated the optical emission properties of the samples by photoluminescence spectroscopy (PL). Figure 6 shows the normalized PL spectra at room temperature for samples A–D. To compare the relative emission efficiency, an unnormalized version of the PL spectra can be found in the Supporting Information (Figure S2). All samples exhibit a broad singular emission peak with a full width at half-maximum (FWHM) between 250 and 330 meV. The quantum well structures in samples C and D show the smallest FWHM. The PL width of these cubic InGaN/GaN quantum wells is comparable to hexagonal InGaN/GaN MQWs for similar emission energies at room temperature.^49−51^

The samples show a large blue-shift for decreasing *t*_In+N_ of more than 400 meV despite the identical indium content of samples A to C; for sample D, the presumably lower indium content can contribute to the emission energy shift. The magnitude of the observed blue-shift of samples A to C is comparable with others reported for cubic InGaN quantum structures with different well thickness.^20,52,53^ Consequently, the blue-shift is consistent with quantum confinement resulting from the developing quantum structures. However, the effect of quantum confinement alone is expected to be less significant, as model calculations using *Nextnano*(54) only show a blue-shift up to 100 meV due to quantum confinement. An additional parameter that could contribute to this blue-shift is compressive strain.^55,56^ The compressive strain affects the band structure of the InGaN material by increasing the bandgap energy, which blue-shifts the PL emission energy. The strain for samples B–D is much higher than that of sample A, with D having the highest strain (Figure S3). This increase in strain is closely linked to the reduction in the quantum well thickness.

## Conclusion

Metal-modulated growth yields a high-quality, fully alloyed cubic InGaN layer as well as c-InGaN/GaN MQWs. The growth mechanism fully explains the emergence of multilayer structures by varying *t*_In+N_ and allows for control of the well width and barrier thickness. The c-InGaN/GaN quantum wells show high quality and abrupt interfaces of its nanostructure, manifested by ToF-SIMS, STEM, and EDX and XRD SL peaks up to sixth order. These multi quantum well structures form naturally and are self-assembled. This is a big advantage over conventionally grown c-InGaN/GaN MQWs, which are challenging to grow with optimal conditions. Furthermore, the purity of the cubic phase of the samples is excellent. This phase purity is assigned to high strain, which the modulated growth method induces. Photoluminescence spectra show the optical emission properties of the samples with a 400 meV large blue-shift appearing for the transition from the alloyed c-InGaN layers to the quantum well samples. This blue-shift is assigned to quantum confinement and strain. All these results show the possibilities and advantages of this metal-modulated method and encourage further investigations on the road toward high-efficiency next-generation optoelectronic applications.

## Supporting Information Available

Atomic force microscopy images with associated RMS values of samples A–D; unnormalized photoluminescence spectra at 290 K of samples A–D; reciprocal space maps of the (−1–13) diffraction peak of c-GaN and c-InGaN for samples A–D (PDF)
