# Supplementary material for: Metal-Modulated Growth of Cubic, Red-Emitting InGaN Layers and Self-Assembled InGaN/GaN Quantum Wells by Molecular Beam Epitaxy
Source: ACS Appl Electron Mater. 2025 Feb 28;7(5):1891–8. doi: 10.1021/acsaelm.4c02174 (PMC11905880; doi:10.1021/acsaelm.4c02174)
Supplement: Supplementary file 1 — el4c02174_si_001.pdf [file el4c02174_si_001.pdf]

Supporting Information: Metal-Modulated Growth of Cubic, Red Emitting InGaN Layers and Self-Assembled InGaN/GaN Quantum Wells by Molecular Beam Epitaxy

Silas A. Jentsch[1], Mario F. Zscherp[1], Vitalii Lider[2], Fabian Winkler[2], Andreas Beyer[2], Jürgen Belz[2], Nicolai M. Gimbel[1], Markus Stein[1], Donat J. As[3], Anja Henss[1], Kerstin Volz[2], Sangam Chatterjee[1], and Jörg Schörmann[1]*

1: Institute of Experimental Physics I and Center for Materials Research, Justus Liebig University Giessen, Heinrich-Buff-Ring 16, D-35392 Giessen, Germany

2: Materials Sciences Center and Faculty of Physics, Philipps-University Marburg, Hans-Meerwein-Strasse 6, D-35032 Marburg, Germany

3: Department of Physics, Paderborn University, Warburger Strasse 100, D-33098 Paderborn, Germany

Atomic force microscopy images for samples A-D

The atomic force microscopy images provide additional insight into the morphology of the four samples. It can be seen that the roughness decreases dramatically from sample A to D.

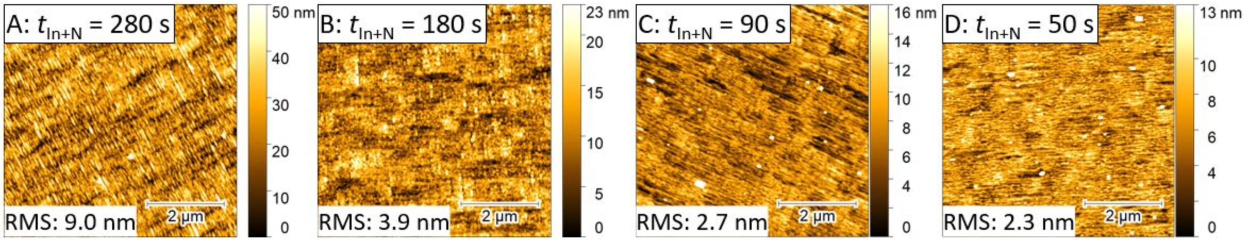

Figure S1. Atomic force microscopy images with associated RMS values of samples A-D with the different t_{In+N}.

Unnormalized photoluminescence spectra at 290 K for samples A-D

These spectra were collected under identical excitation and collection conditions, offering insight into the relative emission efficiency of each sample. The observed decrease in intensity from sample A to sample D is likely related to the decreasing volume of radiative material as the quantum well thickness decreases.

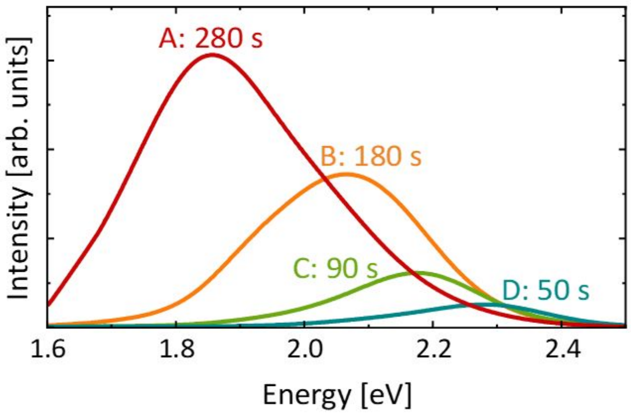

Figure S2. Unnormalized photoluminescence spectra at 290 K of samples A-D with the different $t_{\mathrm{In+N}}$.

Reciprocal space maps of the (−1−13) diffraction peak of c-GaN and c-InGaN for samples A-D

Asymmetric reciprocal space maps of the (−1−13) diffraction peak are required to determine the strain of samples A-D (Figure S3). The position of the c-InGaN peak can be calculated to the in-plane and out-of-plane lattice constants. Using this measured lattice constant and the theoretical relaxed lattice constant, the out-of-plane strain can be determined. The strain can also be estimated visually by examining the proximity of the c-InGaN/SL peaks to the fully relaxed (red dashed line r = 1) or fully strained c-InGaN line (red solid line r = 0). Note that the strain increases from sample A to sample D, with sample A being the most relaxed and sample D being the most strained.

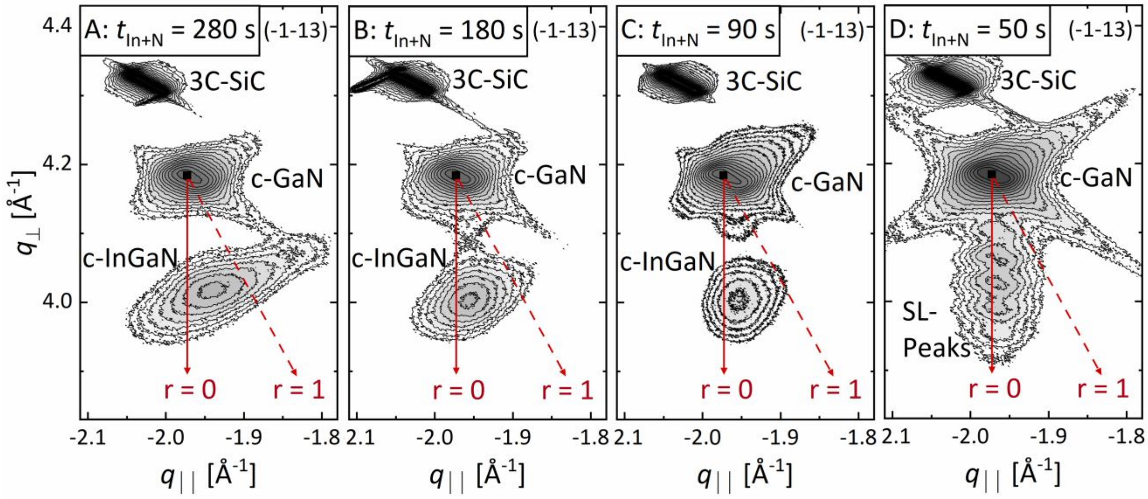

Figure S3. Reciprocal space maps of the (−1−13) diffraction peak of c-GaN and c-InGaN for samples A-D with different t_{In+N}. The red solid line visualizes the possible peak positions for fully strain c-InGaN for different indium contents (relaxation r = 0). The red dotted line does the same for fully relaxed c-InGaN (r = 1).

AUTHOR INFORMATION

Corresponding Author

*Jörg Schörmann: Institute of Experimental Physics I and Center for Materials Research, Justus Liebig University Giessen, Heinrich-Buff-Ring 16, D-35392 Giessen, Germany, Email: joerg.schoermann@exp1.physik.uni-giessen.de
